# Supplementary material for: The high burden of comorbidities in Aboriginal and Torres Strait Islander Australians living with chronic hepatitis B in Far North Queensland, Australia, and the implications for patient management
Source: PLoS One. 2023 Apr 6;18(4):e0284151. doi: 10.1371/journal.pone.0284151 (PMC10079072; doi:10.1371/journal.pone.0284151)
Supplement: S1 Table — (DOCX) [file pone.0284151.s001.docx]

**Supplementary table 1. The annual incidence of the 9 most common cancers in the study region, the availability of screening programme and whether tobacco smoking, alcohol use and obesity are risk factors for the disease (62).**

| Cancer | Annual incidence/100000 population | Screening programme | Smoking as a risk factor | Alcohol as a risk factor | Obesity as a risk factor |
| --- | --- | --- | --- | --- | --- |
| Lung | 32 | +/- ^a^ | + |  |  |
| Prostate | 30 | +/- ^a^ |  |  |  |
| Breast | 24 | + |  | + | + |
| Colorectal | 23 | + | + | + | + |
| Uterus | 22 |  |  |  | + |
| Cervix | 11 | + | + |  |  |
| Oesophagus | 9 |  | + | + |  |
| Stomach | 9 |  | + | + |  |
| Liver | 8 | +/- ^b^ | + | + | + |

^a^ Although screening programmes are available for lung cancer and prostate cancer, they are unlikely to be cost-effective and not presently recommended in the region.

^b^  The cost effectiveness of current national guidelines for HCC screening have yet to be established in the region
