# Supplementary material for: The high burden of comorbidities in Aboriginal and Torres Strait Islander Australians living with chronic hepatitis B in Far North Queensland, Australia, and the implications for patient management
Source: PLoS One. 2023 Apr 6;18(4):e0284151. doi: 10.1371/journal.pone.0284151 (PMC10079072; doi:10.1371/journal.pone.0284151)
Supplement: Attachment — Submitted filename: PONE-D-22-10364R1 Response to reviewers.docx [file pone.0284151.s002.docx]

**Response to reviewers**

We thank the Editorial staff and the reviewer, Dr Ahmed M Elsharkawy, for the time that they have taken to review our manuscript and the very helpful comments that they have made to improve the work. Please find below our point-by-point responses to their comments. For clarity, their comments are presented in blue normal text, while our responses are in black normal text.

Editorial staff comments

When submitting your revision, we need you to address these additional requirements.

1. Please ensure that your manuscript meets PLOS ONE's style requirements, including those for file naming. The PLOS ONE style templates can be found at 
<https://journals.plos.org/plosone/s/file?id=wjVg/PLOSOne_formatting_sample_main_body.pdf> and 
<https://journals.plos.org/plosone/s/file?id=ba62/PLOSOne_formatting_sample_title_authors_affiliations.pdf>.

**Response:** We have reviewed the above templates and believe that our manuscript meets PLOS ONE’s style requirements. Please advise us if there are additional specific changes that we need to make.

2. In your Data Availability statement, you have not specified where the minimal data set underlying the results described in your manuscript can be found. PLOS defines a study's minimal data set as the underlying data used to reach the conclusions drawn in the manuscript and any additional data required to replicate the reported study findings in their entirety. All PLOS journals require that the minimal data set be made fully available. For more information about our data policy, please see <http://journals.plos.org/plosone/s/data-availability>.

Upon re-submitting your revised manuscript, please upload your study’s minimal underlying data set as either Supporting Information files or to a stable, public repository and include the relevant URLs, DOIs, or accession numbers within your revised cover letter. For a list of acceptable repositories, please see <http://journals.plos.org/plosone/s/data-availability#loc-recommended-repositories>. Any potentially identifying patient information must be fully anonymized.

Important: If there are ethical or legal restrictions to sharing your data publicly, please explain these restrictions in detail. Please see our guidelines for more information on what we consider unacceptable restrictions to publicly sharing data: <http://journals.plos.org/plosone/s/data-availability#loc-unacceptable-data-access-restrictions>. Note that it is not acceptable for the authors to be the sole named individuals responsible for ensuring data access.

We will update your Data Availability statement to reflect the information you provide in your cover letter.

**Response:** Unfortunately, we are unable to make our dataset publicly available. This is because the data are obtained from the Queensland State Notifiable Conditions System (NOCS) and data at an individual level are not available publicly because of the Queensland Public Health Act of 2005. However, data are available from the Far North Queensland Human Research Ethics Committee (contact via email FNQ_HREC@health.qld.gov.au) for researchers who meet the criteria for access to confidential data.

Our data Availability Statement might read “Data cannot be shared publicly because of the Queensland Public Health Act of 2005. However, data are available from the Far North Queensland Human Research Ethics Committee (contact via email FNQ_HREC@health.qld.gov.au) for researchers who meet the criteria for access to confidential data.”

3. We note that Figure 1 in your submission contain [map/satellite] images which may be copyrighted. All PLOS content is published under the Creative Commons Attribution License (CC BY 4.0), which means that the manuscript, images, and Supporting Information files will be freely available online, and any third party is permitted to access, download, copy, distribute, and use these materials in any way, even commercially, with proper attribution. For these reasons, we cannot publish previously copyrighted maps or satellite images created using proprietary data, such as Google software (Google Maps, Street View, and Earth). For more information, see our copyright guidelines: <http://journals.plos.org/plosone/s/licenses-and-copyright>.
 
We require you to either (1) present written permission from the copyright holder to publish these figures specifically under the CC BY 4.0 license, or (2) remove the figures from your submission:
 
a) You may seek permission from the original copyright holder of Figure 1 to publish the content specifically under the CC BY 4.0 license.  
 
We recommend that you contact the original copyright holder with the Content Permission Form (<http://journals.plos.org/plosone/s/file?id=7c09/content-permission-form.pdf>) and the following text:
“I request permission for the open-access journal PLOS ONE to publish XXX under the Creative Commons Attribution License (CCAL) CC BY 4.0 (<http://creativecommons.org/licenses/by/4.0/>). Please be aware that this license allows unrestricted use and distribution, even commercially, by third parties. Please reply and provide explicit written permission to publish XXX under a CC BY license and complete the attached form.”
 
Please upload the completed Content Permission Form or other proof of granted permissions as an "Other" file with your submission.
 
In the figure caption of the copyrighted figure, please include the following text: “Reprinted from [ref] under a CC BY license, with permission from [name of publisher], original copyright [original copyright year].”
 
b) If you are unable to obtain permission from the original copyright holder to publish these figures under the CC BY 4.0 license or if the copyright holder’s requirements are incompatible with the CC BY 4.0 license, please either i) remove the figure or ii) supply a replacement figure that complies with the CC BY 4.0 license. Please check copyright information on all replacement figures and update the figure caption with source information. If applicable, please specify in the figure caption text when a figure is similar but not identical to the original image and is therefore for illustrative purposes only.
The following resources for replacing copyrighted map figures may be helpful:
 
USGS National Map Viewer (public domain): <http://viewer.nationalmap.gov/viewer/>
The Gateway to Astronaut Photography of Earth (public domain): <http://eol.jsc.nasa.gov/sseop/clickmap/>
Maps at the CIA (public domain): <https://www.cia.gov/library/publications/the-world-factbook/index.html> and <https://www.cia.gov/library/publications/cia-maps-publications/index.html>
NASA Earth Observatory (public domain): <http://earthobservatory.nasa.gov/>
Landsat: <http://landsat.visibleearth.nasa.gov/>
USGS EROS (Earth Resources Observatory and Science (EROS) Center) (public domain): [http://eros.usgs.gov/#](http://eros.usgs.gov/)
Natural Earth (public domain): <http://www.naturalearthdata.com/>

**Response:** We have attached the Content Permission Form which is signed by the Manager of Data and Systems Support at the Queensland State Government’s Department of Resources. Please let us know if you have any additional concerns. We have also added text below Figure 1 to highlight the origin of data used to construct the map and the software that was used to do it.

“The map was constructed using mapping software (MapInfo version 15.02, Connecticut, USA) using data provided by the State of Queensland (QSpatial). Queensland Place Names—State of Queensland (Department of Natural Resources, Mines and Energy) 2019, available under Creative Commons Attribution 4.0 International licence https://creativecommons.org/licenses/by/4.0/. ‘Coastline and state border–Queensland—State of Queensland (Department of Natural Resources, Mines and Energy) 2019, available under Creative Commons Attribution 4.0 International licence https://creativecommons.org/licenses/by/4.0/.”

Reviewers' comments:

Reviewer's Responses to Questions

**Comments to the Author**

1. Is the manuscript technically sound, and do the data support the conclusions?

The manuscript must describe a technically sound piece of scientific research with data that supports the conclusions. Experiments must have been conducted rigorously, with appropriate controls, replication, and sample sizes. The conclusions must be drawn appropriately based on the data presented.

Reviewer #1: Yes

**Response:** We thank Dr Elsharkawy for reviewing the manuscript and are pleased to hear that he believes that the manuscript is technically sound.

2. Has the statistical analysis been performed appropriately and rigorously?

Reviewer #1: Yes

**Response:** We are happy that Dr Elsharkawy is satisfied with the statistical analysis.

3. Have the authors made all data underlying the findings in their manuscript fully available?

The [PLOS Data policy](http://www.plosone.org/static/policies.action#sharing) requires authors to make all data underlying the findings described in their manuscript fully available without restriction, with rare exception (please refer to the Data Availability Statement in the manuscript PDF file). The data should be provided as part of the manuscript or its supporting information, or deposited to a public repository. For example, in addition to summary statistics, the data points behind means, medians and variance measures should be available. If there are restrictions on publicly sharing data—e.g. participant privacy or use of data from a third party—those must be specified.

Reviewer #1: Yes

**Response:** As noted above**,** data cannot be shared publicly because of the Queensland Public Health Act 2005. Data are available from the Far North Queensland Human Research Ethics Committee (contact via email FNQ_HREC@health.qld.gov.au) for researchers who meet the criteria for access to confidential data. This is because the data are obtained from the Queensland State Notifiable Conditions System (NOCS) and data at an individual level are not available publicly.

4. Is the manuscript presented in an intelligible fashion and written in standard English?

PLOS ONE does not copyedit accepted manuscripts, so the language in submitted articles must be clear, correct, and unambiguous. Any typographical or grammatical errors should be corrected at revision, so please note any specific errors here.

Reviewer #1: Yes

**Response:** We are pleased that Dr Elsharkawy is satisfied with the written presentation of the manuscript.

5. Review Comments to the Author

Please use the space provided to explain your answers to the questions above. You may also include additional comments for the author, including concerns about dual publication, research ethics, or publication ethics. (Please upload your review as an attachment if it exceeds 20,000 characters)

Reviewer #1: In this study, Riddell et al present data on the presence of co-morbidities in a cohort of 236 Indigenous Australians living with chronic hepatitis B in Queensland, Australia. They demonstrate that there is a high burden of co-morbid disease that may drive higher rates of advanced fibrosis and hepatocellular carcinoma and use this to argue that management should be more holistic rather than focused exclusively on management of the viral infection.
It is important to note that current Australian guidelines recommend that all Indigenous Australians above the age of 50 undergo HCC surveillance regardless of fibrosis stage or antiviral therapy status. This underlines the relatively high risk of HCC in this group. However, as highlighted by the authors, this recommendation is fraught with logistical difficulties and finite resources are an issue. However, this disproportionate burden needs to be addressed to try to reduce some of the health inequalities that this group of patients experience; with poorer outcomes in HBV-related HCC in this group being attributed to a higher co-morbid burden and poorer socioeconomic status rather than ethnicity in and of itself.
This study has a clear aim and a clear message and by enlarge the data presented is relevant. There are some points that I think would enhance the quality of the manuscript and help the reader.
**Response:** We thank Dr Elsharkawy again for reviewing the manuscript. We address each of his specific points below.

Other points
1. Do the authors have a sense of how complete screening for HBV is in the geographical area studied? Are the 236 patients self-selecting in that they have better access to healthcare? Having said that the fact that the seroprevalence for HBV was 2.2% suggests that the capture rate was pretty high and this is what is suggested in the discussion.

**Response:** Dr Elsharkawy raises an excellent point. It is certainly likely that some cases of chronic hepatitis B in the region have been missed. However, as hepatitis B is a notifiable disease in the state of Queensland, if a patient records a positive HbsAg or HBV DNA test in any laboratory in the state (public or private), it is automatically reported to the state register. As the state register of notified cases was used to identify cases for this study, we are unlikely to have missed many cases as the region is home to communities that have regular health screening, including HBsAg testing. Indeed, the community prevalence of 2.2% reported in the paper is actually higher than the current estimate of prevalence in Aboriginal and Torres Strait Islander Australians (2.0% reported in reference 1) that is determined using census data.

Even if some cases of chronic hepatitis B have been missed, this is likely to be a relatively small number, and would not be expected to change the main findings of the paper (the high prevalence of comorbidities in the local HbsAg-positive population that increases their risk of liver disease and poor health outcomes generally). We have added some text to the limitations section to address Dr Elsharkawy’s concerns however (lines 376-382).

2. The authors apply a lot of effort into classifying the patients into different HBV disease phases. Whilst this is appreciated, it does not make a significant difference to the central message of the manuscript and the level of detail in this section could perhaps be reduced.

**Response:** We thank Dr Elsharkawy for raising this issue. The staging of disease into different disease phases allows clinicians to determine whether patients are eligible for anti-HBV therapy. Non-cirrhotic patients in the immune tolerant and immune control phases of their chronic hepatitis B infection are not eligible for therapy under current Australian guidelines. Conversely cirrhotic patients, those in the immune escape phase and those in the immune clearance phase for longer than 6 months should be offered therapy if no contraindication exists.

It is estimated that presently only about 53% of Australian patients who are eligible to receive anti-HBV therapy are receiving it (reference 1). In our cohort 38/61 (62%) of those eligible to receive therapy were receiving this medication. An additional 10 patients had been referred for specialist review to commence therapy or had, in shared decision making with their primary provider elected to withhold therapy. In only 13/61 (21%) did the opportunity to discuss an inhiation of therapy appear to have been missed (figure 2 and lines 271-274).

We highlight this point because this shows that the “hepatitis B care” that these patients are receiving is amongst the best in the country. Engagement in care was much higher than the national average (82% versus 23%) and almost 80% of patients eligible for therapy had had treatment discussed with them (versus a national figure of 53%). However, progress in reducing harder endpoints (the incidence of cirrhosis, liver cancer, liver-related mortality, and all-cause mortality) - which after all is the main point of HBV care - will be limited unless other comorbidities are also addressed concurrently (lines 269-280).

3. Figure 4. It would be helpful for the reader to know what the absolute numbers in the overlap areas are. How many patients had all 3 reported risk factors?

**Response:** We agree with Dr Elsharkawy, this is an excellent point. We have amended figure 4 accordingly. There were 19 (8%) individuals in the cohort who had all three risk factors.

4. How do the authors account for the fact that some patients with cirrhosis were not on antiviral therapy despite the need for this being absolutely clearly defined in all the international guidelines?

**Response:** We thank Dr Elsharkawy for highlighting this point. As we describe in figure 2, there were 7/19 patients with known cirrhosis who were not on therapy: 3 were under observation (the risk of flare and decompensation in the setting of poor adherence felt by the clinicians to outweigh the undoubted benefit of this therapy), 1 was not engaged in care, 1 had been referred for specialist review (the primary care provider did not feel confident to start the anti-HBV therapy) and 2 had been apparently missed.

5. Although not essential, it would add to the manuscript to know which antivirals the patients were prescribed and if possible the length that they had been on the agents for. Also, it would help to contextualise matters to know if antiviral medication is fully provided by the State or if the patient has any costs to bear.

**Response:** We thank Dr Elsharkawy for highlighting this point. There were 19 patients receiving tenofovir and 19 patients receiving entecavir. The cost of this medication is fully borne by the State. We have amended the text to highlight this point (lines 133-134 and lines 195-196).

6. How complete was the data on smoking patterns and alcohol consumption? Was it all contemporaneous?

**Response:** We thank Dr Elsharkawy for highlighting this point. The data on smoking and hazardously alcohol consumption was taken from the patient’s electronic medical record, which is a live document. We have amended the methods to reflect this. These records were reviewed to determine if the patients were a current, past, or never smoker. They were also reviewed to determine if there was a history of - or current - hazardous alcohol use (regular consumption of >10 units of alcohol per week or regular binges of greater than 4 units per day) (lines 166-169).

7. The authors should comment on whether the COVID-19 pandemic had any impact on their findings – e.g the number of patients who had one USS in 2020 compared to the fact that they would have been expected to have had a couple if on HCC surveillance.

**Response:** We thank Dr Elsharkawy for highlighting this point. The COVID-19 pandemic did indeed impact on the delivery of care during 2020. We have added text in the discussion to highlight this point (lines 348-349).

8. The authors discuss whether or not HCC surveillance in the studied population is cost effective compared to perhaps investing in bowel cancer or cervical screening. I do not think that their data provides sufficient grounds to justify this statement and this section should be tempered.

**Response:** We thank Dr Elsharkawy for highlighting this point, although we respectfully disagree on this issue. There is significant debate about the cost efficacy of HCC surveillance in metropolitan centres, let alone remote settings where this study was performed.

Approximately 90% of HCC in HbsAg positive patients occur in patients with cirrhosis, many of whom are ineligible for curative therapy. In Australia only 14.5% of non-Indigenous patients and 6.6% of Indigenous patients with HCC are eligible for curative surgery (reference 2).

Providing expert ultrasound services to a population of 236 patients spread over a geographical area of 48,000km^2^, 65% of whom are obese (BMI>30 kg/m^2^) is challenging. Ultrasound surveillance has a sensitivity for detecting HCC at any stage is 78% and 45% if it is at an early stage. However, the sensitivity falls significantly in obese patients 21% in patients with a BMI ≥ 30 kg/m^2^ versus 77% in patients with a BMI <30 kg/m^2^ (<https://www.ncbi.nlm.nih.gov/pmc/articles/PMC6940490/>).

The costs of the HCC screening programme would also need to consider the fact that the nearest CT scanner (for confirmation or exclusion of HCC in a patient with a lesion on ultrasound) is approximately 800 kilometres away, accessible only by commercial flight.

In contrast, bowel cancer screening and cervical cancer screening (both of which are more common that liver cancer in the region), can be performed at home, are acceptable to patients, are unaffected by BMI and have been shown to be highly cost-effective <https://pubmed.ncbi.nlm.nih.gov/34499374/> <https://pubmed.ncbi.nlm.nih.gov/21401458/>

We have expanded the discussion to provide more justification for our stance, which we acknowledge needed more supporting data (lines 337-363).
