# Supplementary material for: The high burden of comorbidities in Aboriginal and Torres Strait Islander Australians living with chronic hepatitis B in Far North Queensland, Australia, and the implications for patient management
Source: PLoS One. 2023 Apr 6;18(4):e0284151. doi: 10.1371/journal.pone.0284151 (PMC10079072; doi:10.1371/journal.pone.0284151)
Supplement: Attachment — Submitted filename: PONE-D-22-10364R2 Response to reviewers.docx [file pone.0284151.s003.docx]

**Response to reviewers**

We thank the Editorial staff and the reviewers for the time that they have taken to review our manuscript and the very helpful comments that they have made to improve the work. Please find below our point-by-point responses to their comments. For clarity, their comments are presented in blue normal text, while our responses are in black normal text.

Journal Requirements:

Please review your reference list to ensure that it is complete and correct. If you have cited papers that have been retracted, please include the rationale for doing so in the manuscript text, or remove these references and replace them with relevant current references. Any changes to the reference list should be mentioned in the rebuttal letter that accompanies your revised manuscript. If you need to cite a retracted article, indicate the article’s retracted status in the References list and also include a citation and full reference for the retraction notice.
**Response:** We have reviewed the reference list and believe that it is complete and correct. We have not, to our knowledge, cited any papers that have been retracted. We have not made any changes to the reference list.

Reviewers' comments:

Reviewer's Responses to Questions

**Comments to the Author**

1. If the authors have adequately addressed your comments raised in a previous round of review and you feel that this manuscript is now acceptable for publication, you may indicate that here to bypass the “Comments to the Author” section, enter your conflict of interest statement in the “Confidential to Editor” section, and submit your "Accept" recommendation.

Reviewer #2: (No Response)

Reviewer #3: All comments have been addressed

Reviewer #4: All comments have been addressed

**Response:** We thank the reviewers for the time that they have taken to review the prior versions of the manuscript and our replies to the concerns of Reviewer 1 (Dr Ahmed M Elsharkawy). We are pleased to hear that they feel that all his comments have been addressed.

2. Is the manuscript technically sound, and do the data support the conclusions?

The manuscript must describe a technically sound piece of scientific research with data that supports the conclusions. Experiments must have been conducted rigorously, with appropriate controls, replication, and sample sizes. The conclusions must be drawn appropriately based on the data presented.

Reviewer #2: No

Reviewer #3: Yes

Reviewer #4: Yes

**Response:** We thank Reviewer 3 and 4 for their positive feedback. However, we are disappointed to hear that Reviewer #2 (Dr. Mohamed Alboraie) does not feel that our work is technically sound. If Dr Alboraie is able to detail any specific concerns that he has, we would be happy to address them.

3. Has the statistical analysis been performed appropriately and rigorously?

Reviewer #2: No

Reviewer #3: Yes

Reviewer #4: Yes

**Response:** We thank Reviewer 3 and 4 for their positive feedback. However, we note that Dr. Alboraie has concerns about our statistical analysis. If Dr Alboraie is able to highlight specific deficiencies, we would be happy to try and remedy them.

4. Have the authors made all data underlying the findings in their manuscript fully available?

The [PLOS Data policy](http://www.plosone.org/static/policies.action#sharing) requires authors to make all data underlying the findings described in their manuscript fully available without restriction, with rare exception (please refer to the Data Availability Statement in the manuscript PDF file). The data should be provided as part of the manuscript or its supporting information, or deposited to a public repository. For example, in addition to summary statistics, the data points behind means, medians and variance measures should be available. If there are restrictions on publicly sharing data—e.g. participant privacy or use of data from a third party—those must be specified.

Reviewer #2: Yes

Reviewer #3: Yes

Reviewer #4: No

**Response:** Unfortunately, the Queensland Public Health Act of 2005 prevents us from including the full dataset in our submission. The same legislation precludes us depositing the dataset in a public repository. However, interested readers can access the complete deidentified dataset by contacting the Far North Queensland Human Research Ethics Committee. We have provided a statement to this effect in our submission: “Data cannot be shared publicly because of the ethical protections of the Queensland Public Health Act of 2005. However, data are available from the Far North Queensland Human Research Ethics Committee (contact via email FNQ_HREC@health.qld.gov.au) for researchers who meet the criteria for access to confidential data”.

5. Is the manuscript presented in an intelligible fashion and written in standard English?

PLOS ONE does not copyedit accepted manuscripts, so the language in submitted articles must be clear, correct, and unambiguous. Any typographical or grammatical errors should be corrected at revision, so please note any specific errors here.

Reviewer #2: No

Reviewer #3: Yes

Reviewer #4: Yes

**Response:** We are heartened that Reviewers 3 and 4 both feel that the language is clear, correct, and unambiguous. Unfortunately, Dr Alboraie does not agree. We would be happy to address any specific concerns that he has if his able to highlight them for us.

6. Review Comments to the Author

Please use the space provided to explain your answers to the questions above. You may also include additional comments for the author, including concerns about dual publication, research ethics, or publication ethics. (Please upload your review as an attachment if it exceeds 20,000 characters)

Reviewer #2: The data gathered and the descriptive analysis done do not support the conclusion made by the authors.

**Response:** We are disappointed to hear that Dr Alboraie does not feel that the data gathered do not support the conclusion that we have drawn.

However, we would contend that we have been able to present data that supports our conclusions.

Our conclusions ( presented in the conclusions section of the abstract) are:

1. Aboriginal and Torres Strait Islander Australians living with chronic HBV in this region of remote Australia have a high engagement with HBV care.

We would contend that the cohort’s engagement in care was high. The most recent analysis of Australia’s chronic hepatitis B cascade of care suggested that only 22.6% of Australians living with chronic hepatitis B are engaged in care (https://hepatitissa.asn.au/blog/hepatitis-b-targets-australia-23-years-behind-on-care-24-on-treatment/ ) The fact that 194/236 (82.2%) of the cohort were engaged in care (almost four times greater than Australia’s national figure and greater than the country’s 2030 target of 50%) suggests that Aboriginal and Torres Strait Islander Australians living with chronic HBV in this region of remote Australia do indeed have a high engagement with HBV care.

2. The majority of individuals eligible for antiviral therapy are receiving it.

61 (31.4%) met national criteria for HBV therapy and 38 (62.2%) - a majority - were receiving it.

3. A significant comorbidity burden increases their risk of cirrhosis, HCC, and premature death.

We would contend that the fact that patients in the cohort had a median (IQR) of 3 (2-4) cardiovascular risk factors, that 142/236 (60.2%) were obese, that 73/236 (30.9%) were current smokers and that 57/236 (24.2%) were drinking alcohol hazardously does represent a significant comorbidity burden. It is hard to argue that this would not be expected to increase the risk of cirrhosis, HCC, and premature death (liver-related and non-liver related) death.

4. It is essential to integrate chronic HBV care with management of these comorbidities - rather than focusing on HBV alone - to achieve optimal health outcomes.

We would argue that this is self-evident. If we don’t also address the patients’ obesity, hazardous alcohol consumption and tobacco use, we will inevitable not achieve optimal health outcomes, no matter how well we manage their hepatitis B.

Reviewer #3: Summary of the research and overall impression
The manuscript discusses the high burden of co-morbidities among the indigenous Australian population and the implication for delivery of care to people living with chronic Hepatitis B. It is hoped that with holistic care, the mortality associated with Hepatocellular carcinoma in this group of people and burden of other associated diseases would be reduced. The paper is well written with a clear aim and sound arguments.
The following points should however be addressed.
Minor areas for improvement
1. The whole discussion on HCC surveillance and its cost-effectiveness, though important, does not appear to emanate from the data provided from the study and indeed is not part of the aims and objectives of the study. I recommend just a brief mention of it. It may appear as part of recommendations for further studies.
2. A few grammatical corrections are needed. For instance, the word “had” should be omitted from line 304.

**Response:** We thank reviewer 3 for the time that they have taken to review our paper and their constructive feedback. We acknowledge that we have not performed a health economic analysis of the currently recommended HCC surveillance strategies in the region and therefore we need to be cautious in our discussion around cost-effectiveness of different approaches. We have therefore added this as a limitation of our study and its potential as a subject for detailed future study (lines 393-396).

However, we would argue that highlighting the burden of comorbidities among people living with chronic hepatitis B in the region is an important focus of the study. It is critical to define the prevalence of comorbidities and their potential impact on HCC incidence as, only then, after the optimisation of their management are we able to determine the incremental value of HCC surveillance.

We do highlight these points in the introduction where we outline the aims of the study. We note that “The optimal approach to reducing HCC-related mortality among Indigenous Australians living with CHB remains incompletely defined” (lines 76-77). We highlight uncertainty about the cost-effectiveness of HCC surveillance in remote Australia (lines 81-83). We also emphasise the importance of optimising the management of comorbidities particularly hazardous alcohol use and obesity (lines 95-96). We then go on to say, “This study was performed to determine the burden of these comorbidities in an effort to inform the delivery of optimal - and holistic - chronic disease care to the people living with CHB in the region” (lines 99-101), which clearly involves the utility – or lack thereof – of HCC surveillance.

We would contend that having identified a very high rate of comorbidity (cardiovascular risk factors and smoking, obesity and hazardous alcohol use) which all had an impact on the 5 most common causes of death in the local Indigenous Australian population and relatively low rates of HCC (only the 9^th^ most common cancer in the region by incidence), we are justified in suggesting that biannual ultrasound surveillance for HCC (across a geographical area of 48,000 km^2^) is unlikely to be a very high value investigation for reducing HCC-related mortality. The region reports approximately 1 case of HCC every year (reference 16). In the year of this study, HCC surveillance did not identify a single HCC in the population.

Indeed, the single case in the cohort with an active, confirmed HCC was diagnosed with multifocal HCC when he presented with ascites; a surveillance ultrasound 8 months prior had been reported as normal. His AFP at diagnosis (2.7 µg/L) was normal. His case was discussed in a multi-disciplinary team meeting with a transplant centre and as he had decompensated disease, no locoregional or systemic options were available to treat the HCC and a palliative approach was recommended (lines 215-222). Although he was two months overdue for his biannual ultrasound, it is unlikely that it would have identified a curable lesion.

We have corrected the typographical error in line 304, omitting “had” from the line. We would be happy to address any other grammatical errors that the Reviewer or Editorial staff highlight.

Reviewer #4: Thanks for the modifications done, it is really an important issue discussing the impact of the comorbidities on HBV and this will have a great impact on the management and follow up of patients.

**Response:** We thank Reviewer 4 again for the time that they have taken to review our paper and for their very positive feedback.
